# Inferior outcome of rotator cuff repair in chronic hemodialytic patients

**DOI:** 10.1186/s12891-019-2597-x

**Published:** 2019-05-13

**Authors:** Kuan-Ting Wu, Wen-Yi Chou, Jih-Yang Ko, Ka-Kit Siu, Ya-Ju Yang

**Affiliations:** 1 Department of Orthopedic Surgery, Kaohsiung Chang Gung Memorial Hospital Medical Center, 123 Ta Pei Road, Niao Sung Dist, Kaohsiung, Taiwan; 2 Department of Medical Research, Kaohsiung Chang Gung Memorial Hospital, Graduate Institute of Clinical Medical Science, Chang Gung University College of Medicine, Kaohsiung, Taiwan

**Keywords:** Rotator cuff, Open repair, End-stage renal disease, Hemodialysis, Shoulder

## Abstract

**Background:**

Repair of rotator cuff tears has yielded excellent functional outcomes in recent decades; however, poor outcomes and dissatisfaction have been noted in specific groups. Spontaneous tendon rupture has been reported in patients receiving long-term hemodialysis owing to alteration of tendon structure, which might impede functional recovery after rotator cuff repair. The purpose of the present study was to compare the clinical outcomes between hemodialysis and non-hemodialysis patients after rotator cuff repair.

**Methods:**

We retrospectively reviewed patients who underwent mini-open rotator cuff repair from Jan 2013 to Jan 2017. A total of 14 patients under chronic hemodialysis (HD) were matched to non-hemodialysis (NHD) patients at a 1:2 ratio according to age, gender, tear size, severity of fatty infiltration and history of diabetes. Pre- and post-operative functional outcome was assessed using the simple shoulder test (SST), American Shoulder and Elbow Surgeons (ASES), Shoulder Rating Scale of the University of California at Los Angeles (UCLA) and visual analog scale (VAS) scores. Clinical functional outcome at the last follow-up was adopted for comparison of the HD and NHD groups.

**Results:**

A total of 42 patients were enrolled in this comparative study, with a mean age of 66.64 ± 1.68 years in the HD group and 65.71 ± 5.40 years in the NHD group. At the final clinical assessment, the post-operative functional outcome was significantly improved in both groups (*p* < 0.001). However, the functional outcome of the HD group was significantly inferior to that of the NHD group in terms of the SST score (6.50 ± 2.24 vs 9.39 ± 1.87, *p* < 0.001), ASES score (63.17 ± 15.93 vs 86.96 ± 11.43, p < 0.001), UCLA score (20.14 ± 7.71 vs 29.82 ± 5.08, p < 0.001) and VAS score (3.00 ± 0.96 vs 1.21 ± 1.03, p < 0.001).

**Conclusion:**

The improvement of pain and functional improvement of long-term hemodialysis patients were inferior to those of patients without hemodialysis after mini-open rotator cuff repair.

## Background

Rotator cuff tear is a common disease that causes shoulder pain, disability and weakness. In a systemic review including 6112 shoulders, the prevalence of rotator cuff disease increased in the aging population, being 9.7% in those under 20 years of age and 62% in patients older than 80 [1]. Since the emergence of improved diagnostic imaging and surgical techniques, rotator cuff repair has become the standard procedure for symptomatic tears, and a satisfaction rate of 80 to 95% has been reported after rotator cuff repair via the open or arthroscopic technique after a follow-up duration of 2–4 years [2–6]. In studies with a follow-up duration of more than 10 years, researchers found that functional improvement was maintained, and nearly 90% of patients were satisfied with the outcome after rotator cuff repair [7, 8].

Despite the high satisfaction rate for rotator cuff repair, patients with inferior outcomes and dissatisfaction do exist. In the 1990s, Harryman et al. [7] reported greater recurrent defects after rotator cuff repair in older patients and in patients who had a larger defect, with an approximate re-tear rate in isolated supraspinatus tendons of 20% and in patients with multiple tendon involvement of more than 50%. In a long-term prospective study of 105 shoulders, Cofield et al. [8] identified tear size as an important prognostic factor of functional outcome and satisfaction. Goutallier et al. [9] developed a 5-stage grading system to quantify the fatty degeneration of rotator cuff muscles. A greater recurrent tear rate was found in patients with fatty degeneration higher than grade 1, and a poorer prognosis was found to be related to fatty infiltration of the supraspinatus and infraspinatus muscles in subsequent studies [10, 11].

In addition to morphologic factors, comorbidities have also been reported to result in inferior functional outcomes of rotator cuff repair. In a review of the literature, diabetes was identified as an important risk factor for tenosynovitis and joint stiffness owing to a high proportion of disorganized tendon fibers [12, 13]. In addition, chronic kidney disease has been suggested to be a risk factor for several complications in orthopedic surgery [14], especially in hemodialysis patients [15]. In a study of musculoskeletal complications in hemodialysis patients, an occurrence rate of almost 80% for supraspinatus tendinitis was found in patients with chronic hemodialysis [16]. Furthermore, the reported incidence of rotator cuff rupture ranges from 9 to 33% among chronic hemodialysis patients [17–19]. Spontaneous major tendon rupture has been noted in patients under hemodialysis, although the exact pathomechanism remains controversial [20]. It is believed that loss of tendon elasticity and weakening of the structure might be the major factors in spontaneous tears. Other factors related to spontaneous tears in chronic hemodialysis patients include malnutrition, β2-amyloidosis, chronic acidosis and uremic toxins [21, 22]. Therefore, we postulated that alteration of tendon structure might impede functional recovery after rotator cuff repair in chronic hemodialysis patients.

The purpose of the present study was to compare the clinical outcome between hemodialysis and non-hemodialysis patients after rotator cuff repair.

## Materials and methods

From January 2013 to January 2017, patients who underwent mini-open rotator cuff repair for a complete tear were systematically reviewed. This study was approved by our Institutional Review Board. The diagnosis of rotator cuff tear was initially made on clinical presentation and following plain radiographs, and further confirmed by either soft tissue sonography or magnetic resonance imaging (MRI). Patients who underwent rotator cuff repair were those with a chronic, symptomatic full-thickness tear who failed to respond to oral medicine and physiotherapy for at least 3 months. The exclusion criteria of the present analysis included surgery for a partial rotator cuff tear, advanced glenohumeral arthritis, traumatic rotator cuff tear, history of humeral head fracture and history of septic arthritis. Mini-open repair was favored over arthroscopic repair for hemodialysis patients in order to minimize traction-related complications, which may jeopardize the arteriovenous shunt or the artificial shunting system. Besides, mini-open rotator cuff repair requires less anesthesia and a shorter surgical duration, which may allay concerns in chronic hemodialysis patients. Therefore, patients who underwent arthroscopic rotator cuff repair were also excluded from this retrospective comparative analysis.

### Pre-operative parameter evaluations

The pre-operative parameters evaluated in this study included age, gender, smoking status, diabetes mellitus, tear size and fatty infiltration by the Goutallier classification method [9]. The tear size was initially investigated by soft tissue sonography or MRI and was confirmed by intra-operative findings. Cofield et al. [8] classified the tear size as follows: small tear, < 1 cm; medium tear, 1-3 cm; large tear, 3-5 cm; massive tear,> 5 cm. Irreparable tears were recognized as massive tears, the remnant of the tendon being irreducible to the junction of the cartilage and the rough area of greater tuberosity of the humerus, which would render marginal converging repair not feasible, despite extensive release having been performed. Fatty infiltration was classified into grade I (completely normal muscle without any fatty streaks), grade II (muscle contains some fatty streaks, less than 50%), grade III (equal amounts of fat and muscle), and grade IV (more fat than muscle is present) [11].

### Pre- and post-operative functional outcome evaluation

The subjective pain score was recorded on a visual analog scale (VAS). Shoulder functional assessment was conducted via a simple shoulder test (SST) [23]; the American Shoulder and Elbow Surgeons (ASES) score was measured [24], and the Shoulder Rating Scale of the University of California at Los Angeles(UCLA) was recorded. The SST reflects the status of the shoulder in functional terms rather than via range of motion, radiographs or muscular strength testing. The ASES score includes patient self-evaluation and physical assessment sections, which minimizes assessment bias from physicians only. Although the UCLA shoulder score was initially developed for patients with shoulder arthritis undergoing total shoulder arthroplasty, it has been widely-used for specific disorders, such as following treatment after rotator cuff repair [25]. All the scores were obtained before surgery and at each post-operative outpatient follow-up. Clinical follow-up was performed at the 1st, 3rd, 6th and 12th months post-operatively, and continued once a year after the 2nd post-operative year. The clinical functional outcome at the last follow-up was adopted for comparison with the pre-operative status.

### Surgical technique

**Fig. 1:**
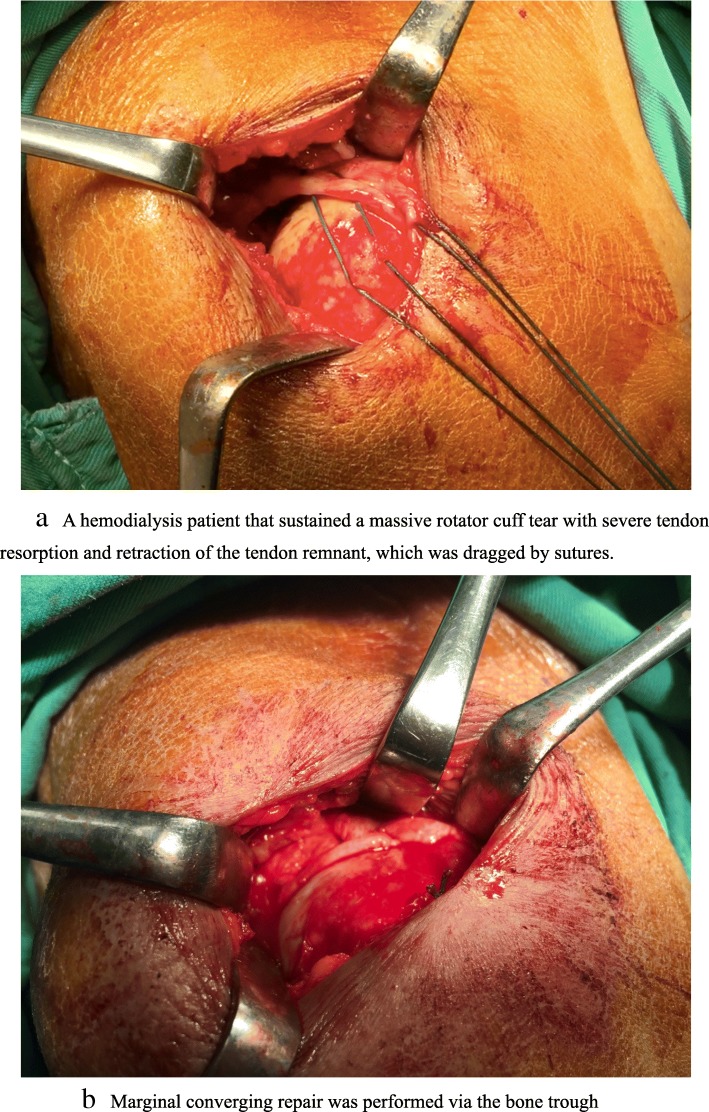
**a** A hemodialysis patient that sustained a massive rotator cuff tear with severe tendon resorption and retraction of the tendon remnant, which was dragged by sutures. **b** Marginal converging repair was performed via the bone trough

Mini-open rotator cuff repair was performed under general anesthesia. The approach began from the anterior border of the acromion, with a 4–5-cm incision. The deltoid muscle was split through blunt dissection along the muscle fibers, and then a small part of the anterior deltoid was detached from the anterior acromion in order to perform acromioplasty with an osteotome, and the undersurface of the acromion was shaved to obtain a smooth surface. The surgical field was exposed with muscle retractors and partial subacromial bursectomy was performed accordingly. After evaluation of tear size and quality of the tendon remnant, soft tissue release was performed to mitigate the tension of the tendon. Then, the tendon was repaired with No. 2 ethibond (Ethicon Inc., Somerville, NJ, USA) using simple side-to-side tendon repair, or horizontal mattress transosseous repair via the bone trough if the rotator cuff could be reduced into an anatomic position, or via the rough area of greater tuberosity, which was recognized as marginal converging repair (Fig. 1a and b). In the case of an irreparable tear, partial repair was performed in order to secure re-attachment of the infraspinatus to the posterior side of greater tuberosity and/or the subscapularis of lesser tuberosity. Finally, the split anterior deltoid muscle was repaired and the wound closed in layers.

### Post-operative rehabilitation

The shoulders operated upon were protected with abduction pillows during daily activity for 6 weeks. Passive exercise was initiated on the first post-operative day via pendulum exercise. Low-grade passive forward flexion and abduction were carried out in the first post-operative week under tolerable pain. Active motion and rotational motion were initiated 4 weeks post-operatively for larger tears or smaller and 6 weeks post-operatively for massive and irreparable tears. Intermittent muscle strengthening was initiated from the 7th post-operative week.

### Statistical analysis

The demographic data are presented as the mean ± standard deviation for continuous variables and a percentage for discrete variables with descriptive statistical analysis. Propensity score matching analysis was conducted to control potential confounding factors. The Kolmogorov-Smirnov test was used to test the normality of the data. The Chi-square test was used to compare categorical variables, and the independent t test, paired t test and Wilcoxon signed ranked test were used for comparison of continuous variables between groups. A *p*-value of less than 0.05 was considered significant. All statistical analyses were performed using SPSS software V.21 (SPSS Inc. Chicago, IL, USA).

## Results

**Fig. 2:**
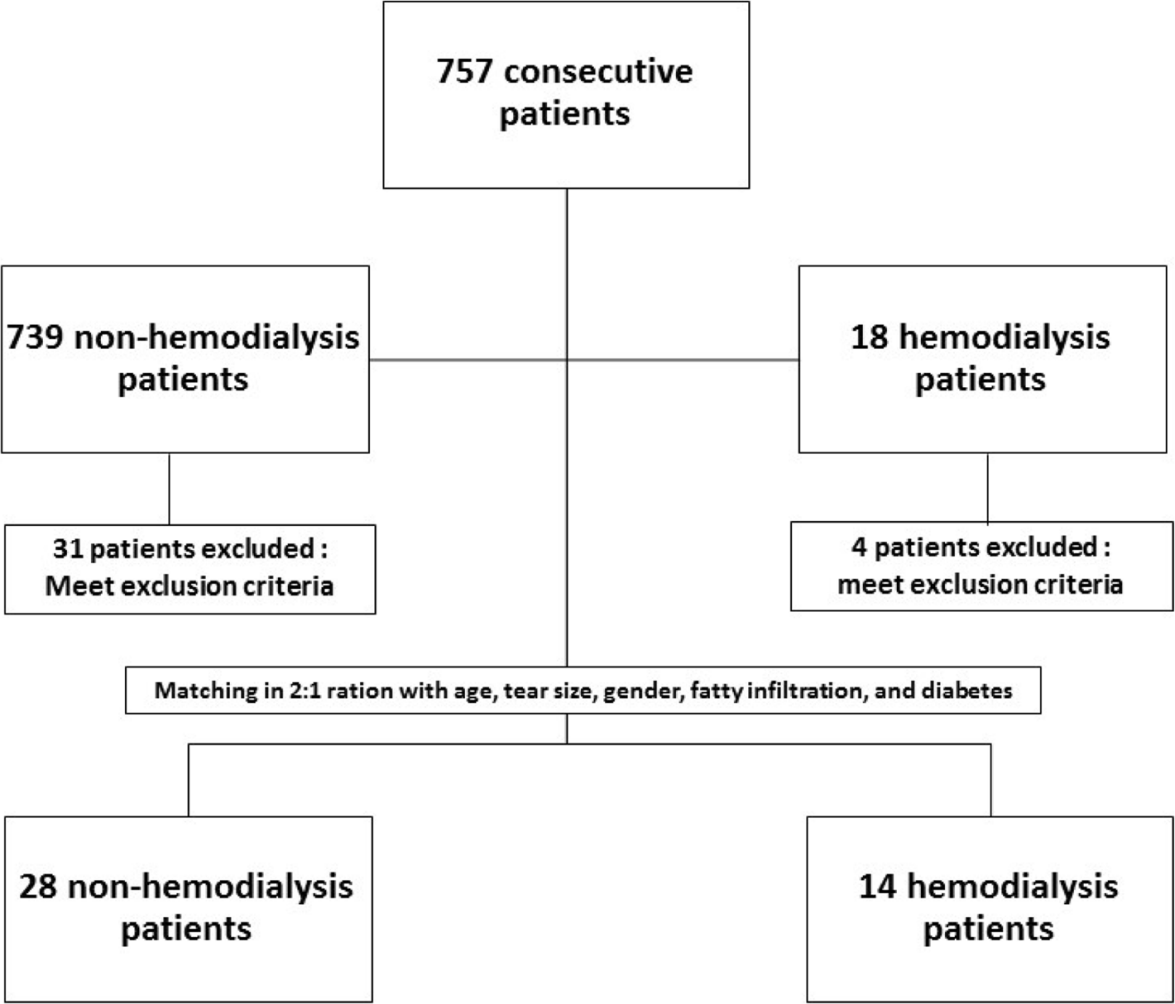
Flowchart of enrollment of patients undergoing mini-open rotator cuff repair from Jan 2013 to Jan 2017

From January 2013 to January 2017, 757 symptomatic rotator cuff tears were treated via mini-open rotator cuff repair. A total of 14 patients were identified as having end-stage renal disease (ESRD) and had been under regular hemodialysis for more than 10 years. In order to minimize the effects of confounders on the functional outcome after rotator cuff repair, we matched the hemodialysis (HD) patients to non-hemodialysis (NHD) patients at a 1:2 ratio according to age, gender, tear size, severity of fatty infiltration and history of diabetes. A total of 42 patients were enrolled in this comparative study (Fig.2), and were classified into the HD group and the NHD group, with a mean age of 66.64 ± 1.68 years and 65.71 ± 5.40 years, respectively. As the groups were matched based on age, gender, diabetes, tear size and fatty infiltration, there were no significant differences in these variables between the HD and NHD groups (Table 1). The mean follow-up duration was 21.5 ± 18.11 months (range, 6–72) in the HD group and 20.11 ± 9.10 months (range, 6–38) in the NHD group (*p* = 0.740).

**Table 1:** Patient demographic data

	NHD *N* = 28	HD *N* = 14	*P* value
Age (Mean ± SD, years)	65.71 ± 5.40	66.64 ± 1.68	0.621
Gender (Male/Female)	20/8	9/5	0.637
Diabetes	0	0	1.000
Smoking	1	0	1.000
Tear size			0.763
Medium(1-3 cm)	8(28.5%)	2(14.3%)	
Large(3-5 cm)	6(21.4%)	5(35.7%)	
Massive(> 5 cm)	7(25%)	3(21.4%)	
Irreparable	7(25%)	4(28.6%)	
Goutlallier classification			1.000
Grade I	0	0	
Grade II	16	8	
Grade III	0	0	
Grade IV	12	6	

Regarding pre-operative shoulder function, the SST score was lower (NHD vs HD = 4.39 ± 1.49 vs 3.36 ± 1.55, *p* = 0.043) in the HD group, but the ASES score (NHD vs HD = 37.03 ± 10.43 vs 30.80 ± 9.51, *p* = 0.056), UCLA score (NHD vs HD = 11.39 ± 3.68 vs 9.93 ± 3.56, *p* = 0.226) and VAS score (NHD vs HD = 7.71 ± 0.60 vs 7.50 ± 0.52, *p* = 0.362) were not statistically significantly different between the two groups (Table 2). At the final clinical assessment, the post-operative functional outcome had improved significantly in both groups (*p* < .001). However, the functional outcome of the HD group was significantly inferior to that of the NHD group in terms of SST score (NHD vs HD = 9.39 ± 1.87 vs 6.50 ± 2.24, *p* < 0.001), ASES score (NHD vs HD = 86.96 ± 11.43 vs 63.17 ± 15.93, p < 0.001), UCLA score (NHD vs HD = 29.82 ± 5.08 vs 20.14 ± 7.71, p < 0.001) and VAS score(NHD vs HD = 1.21 ± 1.03 vs 3.00 ± 0.96, p < 0.001).

**Table 2:** Comparative functional assessment

	NHD N = 28	HD N = 14	*P* value
	Mean(SD)	Mean(SD)	
SST			
Before surgery	4.39(1.49)	3.36(1.55)	0.043
After surgery	9.39(1.87)	6.50(2.24)	< 0.001
*P* value	< 0.001	0.001	
ASES			
Before surgery	37.03(10.43)	30.80(9.51)	0.056
After surgery	86.96(11.43)	63.17(15.93)	< 0.001
*P* value	< 0.001	< 0.001	
UCLA			
Before surgery	11.39(3.68)	9.93(3.56)	0.226
After surgery	29.82(5.08)	20.14(7.71)	< 0.001
*P* value	< 0.001	< 0.001	
VAS			
Before surgery	7.71(0.60)	7.50(0.52)	0.362
After surgery	1.21(1.03)	3.00(0.96)	< 0.001
*P* value	< 0.001	0.001	

*SST* Simple shoulder test, *ASES* American Shoulder and Elbow Surgeons score, *UCLA* University of California Los Angeles shoulder score, *VAS* Visual Analogue Scale

## Discussion

In the present study, we performed detailed functional outcome evaluation in hemodialysis and non-hemodialysis patients who underwent rotator cuff repair, and compared the outcome between groups. Before surgery, the hemodialysis patients had relatively lower SST scores (*p* = 0.043) than the non-hemodialysis patients, which implied poor daily activity functioning; otherwise, there were no significant differences in terms of the ASES score, UCLA score or VAS score (Table 2). After the surgery, the patients in the HD group presented an inferior final functional outcome in terms of the SST score (*p* < .001), ASES score (p < .001) and UCLA score (*p* < 0.001) than those in the NHD group. A relatively poor outcome was also noted in terms of pain relief (HD:NHD = 3.00:1.21, p < 0.001), although significant improvement was attained after surgery.

Sauerbrey et al. [26] reported good to excellent functional outcomes after mini-open rotator cuff repair, with improvement in the ASES score from 52 to 89 on average. Another study demonstrated a 93% good to excellent outcome on the UCLA rating scale in 69 shoulders following mini-open rotator cuff repair [27]. A recent comparative study between arthroscopic repair and mini-open rotator cuff repair using the SST score as the primary outcome measure reported a mean SST score of 10.9 at the 2-year follow-up point in the mini-open group [28]. The present study demonstrated that the functional outcome was in line with the results reported in the literature in the non-hemodialysis group, but the improvement of pain and functional improvement in the chronic hemodialysis patients who underwent rotator cuff repair were inferior to those in the non-hemodialysis patients. In other words, ESRD with long-term hemodialysis is a relatively poor prognostic factor for rotator cuff repair.

Several prognostic factors for rotator cuff healing after surgical repair have been proposed, such as fatty degeneration, muscle atrophy, tear size, and smoking [8, 10, 29, 30]. In the literature, tendon rupture was first described in patients with chronic kidney disease in 1949 by Steiner and Palmer [31]. Spontaneous rupture of multiple tendons has been reported, especially on the quadriceps, patella and triceps tendon, and a longer duration of hemodialysis is known to be one of the most important risk factors owing to weakening of tendon structures [22, 32]. Therefore, it is postulated that comorbidity, such as chronic kidney disease with long-term hemodialysis, is one of the important prognostic factors affecting the outcome of rotator cuff repair and the overall functional outcome. The results of the present analysis verified an inferior surgical outcome in hemodialysis patients. To the best of our knowledge, this was the first study to reveal the comparative outcomes of rotator cuff repair in hemodialysis and non-hemodialysis patients.

β2 microglobulin (β2M) is expressed on nucleated cells and excreted by the kidneys. When the infiltration rate of glomerular decreases, the serum level of β2M is elevated [33]. In hemodialysis patients, serum β2M cannot pass through the cellulose dialysis membrane, and the accumulated β2M tends to be deposited in the musculoskeletal system, especially in the bones, ligaments, tendons, synovium and cartilage [34]. In a study of sonographic features of shoulder joints in hemodialysis patients, Kamel et al. [19] demonstrated that the serum level of β2M was significantly correlated with a thickened supraspinatus tendon, tear of the supraspinatus tendon and humeral head erosion. In addition, irregularity and thickening were also found in the supraspinatus tendon in patients with amyloid accumulation on MRI [35]; this is postulated to be closely related to and interfere with the outcome of tendon repair.

Alteration of tendon structure results in a reduction of elasticity of the tendon [36], and might impede functional recovery after rotator cuff repair in patients with hemodialysis. In other ways, electrolyte and hormone imbalances as a result of kidney failure will change the environment surrounding the tendon and the tendon-bone junction. First, collagen is replaced by elastin, which results in elastosis owing to long-term acidosis in dialysis patients [37]. Second, hyperparathyroidism, which is a classic complication of ESRD in response to phosphorous retention, is an important contributing factor of weakened tendons. Stimulation of osteoclast activity by hyperparathyroidism is prominent around subtendinous sites and results in weakening of the bone cortex [22]. In 2001, Rodeo et al. [38] reported that osteoprotegerin improves stiffness at the healing tendon-bone junction due to inhibition of osteoclast activity. On the other hand, application of receptor activator of nuclear factor-kappa B ligand (RANKL) impaired bone ingrowth and impeded the tendon healing process in a rabbit model. In a clinical study, Chung et al. [30] found that bone mineral density (BMD) is an independent risk factor for rotator cuff healing after arthroscopic repair in a multivariate analysis. Hyperparathyroidism-related renal osteodystrophy is similar to the effect of osteoporosis on tendon healing. In addition, dialysis-related amyloidosis has a high proportion of shoulder involvement [18], Konishiike et al. [39] reporting a 48% incidence of shoulder pain in patients who have received dialysis for an average of nine years. Non-fatty infiltration in the rotator interval was identified by Kerimoglu et al. [40], and has a strong correlation with constraint of shoulder internal rotation, external rotation and abduction motion. These contributing factors to tendon rupture may play roles in the tendon healing process, and even in healed tendons in patients with hemodialysis.

Our study had some limitations. First, the retrospective analysis obtained no pathologic proof of the amyloid deposition or alteration of tendon structures regarded as contributing factors to poor rotator cuff tendon healing. Second, insufficient post-operative soft-tissue imaging, such as MRI or soft tissue sonography, to assess tendon healing weakened the strength of the comparison, although the functional outcome was the major concern in the post-operative patients. Third, even though power analysis was sufficient for the statistical difference in the outcome measurement, the limited number of hemodialysis patients in this study may also have weakened this clinical result.

## Conclusion

The present comparative analysis revealed that improvement in pain and functional improvement in chronic hemodialysis patients who underwent mini-open rotator cuff repair were inferior to those of non-hemodialysis patients, although significant functional improvement was obtained after surgery in both groups. In other words, ESRD with long-term hemodialysis is a relatively poor prognostic factor for rotator cuff repair.

## Abbreviations

ASES: American Shoulder and Elbow Surgeons score
ESRD: End stage renal disease
HD: Hemodialysis
MRI: Magnetic resonance imaging
NHD: Non-hemodialysis
RANKL: Receptor activator of nuclear factor-kappa B ligand
SST: Simple shoulder test
UCLA: Shoulder Rating Scale of the University of California at Los Angeles
VAS: Visual analog scale
β2M: β2 microglobulin
